# Individual movements and contact patterns in a Canadian long-term care facility

**DOI:** 10.3934/publichealth.2018.2.111

**Published:** 2018-05-09

**Authors:** David Champredon, Mehdi Najafi, Marek Laskowski, Ayman Chit, Seyed M. Moghadas

**Affiliations:** 1 Agent-Based Modelling Laboratory, York University, Toronto, ON M3J 1P3, Canada; 2 Department of Mechanical & Industrial Engineering, Faculty of Applied Science & Engineering, University of Toronto, Toronto, ON M5S 3G8, Canada; 3 Schulich School of Business, York University, Toronto, Ontario, Canada M3J1P3, Canada; 4 Sanofi Pasteur, Swiftwater, PA, USA, and Leslie Dan Faculty of Pharmacy, University of Toronto, Toronto, ON M5S 3G8, Canada

**Keywords:** long-term care facility, mobility, contact network, RFID

## Abstract

Contact networks of individuals in healthcare facilities are poorly understood, largely due to the lack of spatio-temporal movement data. A better understanding of such networks of interactions can help improve disease control strategies for nosocomial outbreaks. We sought to determine the spatio-temporal patterns of interactions between individuals using movement data collected in the largest veterans long-term care facility in Canada. We processed close-range contact data generated by the exchange of ultra-low-power radio signals, in a prescribed proximity, between wireless sensors worn by the participants over a two-week period. Statistical analyses of contact and movement data were conducted. We found a clear dichotomy in the contact network and movement patterns between residents and healthcare workers (HCWs) in this facility. Overall, residents tend to have significantly more distinct contacts with the mean of 17.3 (s.d. 3.6) contacts, versus 3.5 (s.d. 2.3) for HCWs (*p*-value < 10^−12^), for a longer duration of time (with mean contact duration of 8 minutes for resident-resident pair versus 4.6 minutes for HCW-resident pair) while being less mobile than HCWs. Analysis of movement data and clustering coefficient of the hourly aggregated network indicates that the contact network is loosely connected (mean clustering coefficient: 0.25, interquartile range 0–0.40), while being highly structured. Our findings bring quantitative insights regarding the contact network and movements in a long-term care facility, which are highly relevant to infer direct human-to-human and indirect (i.e., via the environment) disease transmission processes. This data-driven quantification is essential for validating disease dynamic models, as well as decision analytic methods to inform control strategies for nosocomial infections.

## 1. Introduction

Infectious diseases inflict a significant health and economic burden in hospitals and long-term care facilities. In particular, nosocomial infections (e.g., influenza, pneumonia, gastrointestinal illness, urinary tract infections) have enormous impact on healthcare systems in terms of costs and patient outcomes, incurring billions of dollars every year only in the developed world [1]. Controlling outbreaks in these population settings is particularly challenging, as the source of infection is often unknown. Furthermore, possible congregation during daily activities creates an environment for repeated exposures to infection, which can facilitate disease spread in vulnerable individuals with underlying health conditions. However, little is known about the complex network of interactions in this type of environment. Data-driven studies to address this knowledge gap and quantify the dynamics of contact and movement patterns could greatly enhance our understanding of institutional outbreaks and responses.

Quantification of contact patterns derived from movements and interactions among individuals is critical to infer the potential transmission routes of infectious diseases. Having knowledge of such patterns is important for parameterizing models of disease spread in order to evaluate control and preventive measures and inform clinical decision-making on the most effective and cost-effective interventions. A number of studies have collected and analyzed data pertaining to individuals' movement and contact patterns using various technologies in different settings (for example, large gatherings [2], schools [3],[4], hospitals [5],[6], offices [7]). Here we present our efforts in this direction to analyze close-range contact data collected using Internet of Things (IoT) technologies in a Canadian long-term care facility (LTCF). We hypothesized that the individual links in this LTCF are sparsely connected, but the network of interactions is highly structured.

As shown in our recent study [8], the use of movement and contact data to parameterize transmission dynamic and decision analytic models, instead of assuming random mixing, can lead to substantially different predictions and estimates of the effect of disease control measures. While making such data accessible to the research community (in a compressed and anonymized form), we provide a detailed analysis of movement signals collected through wearable sociometric tags in this LTCF. We identify challenges, gaps, and potential policy implications of utilizing movement data, and suggest ways to improve data collection processes and pertinent technologies to inform future efforts and, in particular, modeling studies.

## 2. Materials and methods

### 2.1. Population setting

We collected movement data in the largest veterans LTCF in Canada, located at the Sunnybrook Health Sciences Centre, Toronto. For data collection, the wearable sociometric tags and radio frequency identification (RFID) readers were distributed in two main sections of this facility, with a total of 50 rooms, and 19 service locations and public areas. The total number of staff working in this facility was 64 in all shifts and the total number of residents was 52. Participation by staff members and residents in the data collection was voluntary. The participation rate was 63% (40 individuals) for the staff and 36% (19 individuals) for the residents. A number of residents were cognitively impaired or had physical disabilities, requiring constant monitoring had they worn sociometric badges for data collection. It was therefore not advisable for them to participate in the study, which resulted in a relatively low participation rate.

### 2.2. Data collection

Ethics approval was obtained from Ethics Research Boards of York University and Sunnybrook Health Sciences Centre. No personal or health information data were collected from study participants. Prior to data collection, informed consent was obtained from staff and patients (and when required from substitute decision makers for residents). Contact data were collected using wearable wireless tags that exchange ultra-low-power radio signals packets in a peer-to-peer fashion to monitor the location and proximity of individuals. With this technology, contact is—in theory—detected when two individuals are in close proximity (e.g., within 1.5 meters) and facing each other. In addition to tagging individuals, some locations within the LTCF, including corridors and residents' rooms, were equipped with static marker tags. This way, we were able to recognize the presence and the duration of stay for each individual in a location where static marker tags were installed (see Supplementary material for more details). Data collection was carried out in two distinct periods (i.e., December 2015 and March 2016), each for a duration of 14 days. The first period (December 2015) was considered as a testing attempt in order to identify potential problems in the process of data collection (e.g., participants' behaviors or technical failures). Our analysis presented here is based on the second period of data collection, which was used to parameterize an agent-based model of influenza transmission dynamics in the LTCF and evaluate the effect of different intervention strategies [8].

### 2.3. Data processing

Recoded proximity of sociometric tags formed two large time-sorted datasets, each of an approximate size 37 GB with the resolution of milliseconds for the duration of the study. The first dataset includes the time history of locations where both moving and static tags were detected by the RFID readers. In this dataset, each entry for a tag includes time, identification number of the tag, and received signal power. The Received Signal Strength Indicator (RSSI) value, determined when a static reader detects signals from an individual's tag, has a reverse relationship with the distance of the tag from the reader. Since the location of the readers were fixed and known, a relative distance of the tag and the reader in a measurement of signal strength could be identified. We also used fine locating algorithms based on geometrical modeling and discretization of the entire long-term care facility. More than one marker tag was used in corridors and areas that have complex physical structures. Since the range of a marker tag radio signal is always larger than the extent of the assigned location, an individual tag would be recognized to be in more than one location at the same time. Thus, we utilized geometrical information in the form of a grid model to locate individuals accurately and avoid multiple-location interpretations. To this end, we built a radio space considering the locations of fixed marker tags as anchors and used trilateration algorithm to estimate the locations of moving tags following the method described in previous studies [9],[10] (Supplementary material).

The second dataset consists of close contacts captured between two individuals' tags, where time of contact, identification numbers of the two tags, and received signals power were recorded. Continuous interaction of the tags with the RFID readers helped to identify interferences and remove incorrect estimations. Unused and misplaced tags were also identified and their records were filtered out from the datasets. In order to reduce the datasets to a manageable size for the analysis presented here, we performed a temporal aggregation and changed the time resolution to one second. Further decrease in the file size of the location dataset was made through an event-based approach. Consequently, a new dataset was formed that only included the changes in location for each tag and the time of that change. Therefore, when a tag was not moving for a long time-duration (e.g., hours), it has only one record rather than several similar records in time. This also helped to perform temporal coarsening of the location dataset without discarding relevant data.

Although the participants were anonymous in the entire data collection process, their roles in the facility were recorded and formed an indexed table with the identification number of the assigned tag. Using this table, we were able to distinguish between healthcare workers and residents activities, and study their interactions separately and together.

Our analysis aims to present the main characteristics of contact and movement patterns observed in the facility over the period of data collection. We studied the distribution of the number and duration of contacts as well as the distribution of time spent near static tags, which give an indication of the spatial movements of individuals. We also performed a simple analysis about the topology of the contact network by calculating the global clustering coefficient (also called transitivity). This coefficient is the average of the fraction of possible interconnections between individuals (i.e., a node) in a network. This fraction for each node is calculated by 2N / (k (k−1)), where N is the number of links between neighbours of a node, and k is the degree of the node, representing the number of links to other nodes. The average of these ratios for all nodes in a network determines the global clustering coefficient [11]. One can interpret its value (which is ranged between 0 and 1) as the probability that neighbours of an individual contact each other given that they are already in contact with the individual (i.e., making triangles of connections).

## 3. Results

Initial analysis of the data revealed significant signal noises on the first and last days of the data collection period, largely due to the sensor tags being distributed and collected from participants. Hence, we chose to disregard these two days in the analysis and considered recorded signals from March 17, 00:01 am to March 28, 12:00 pm (hereafter referred to as the “study period”).

### 3.1. Frequency and characteristics of the contacts

The total number of tags broadcasting recorded signals during daily activities was nearly constant for the residents over the study period, whereas, as expected, it decreased significantly for HCWs during weekends (Figure S2). All tags assigned to the participating residents recorded at least 10 hours of daily between-individual contacts. Among the participating HCWs, 67% recorded at least 1 hour of between-individual contacts.

**Figure 1.**
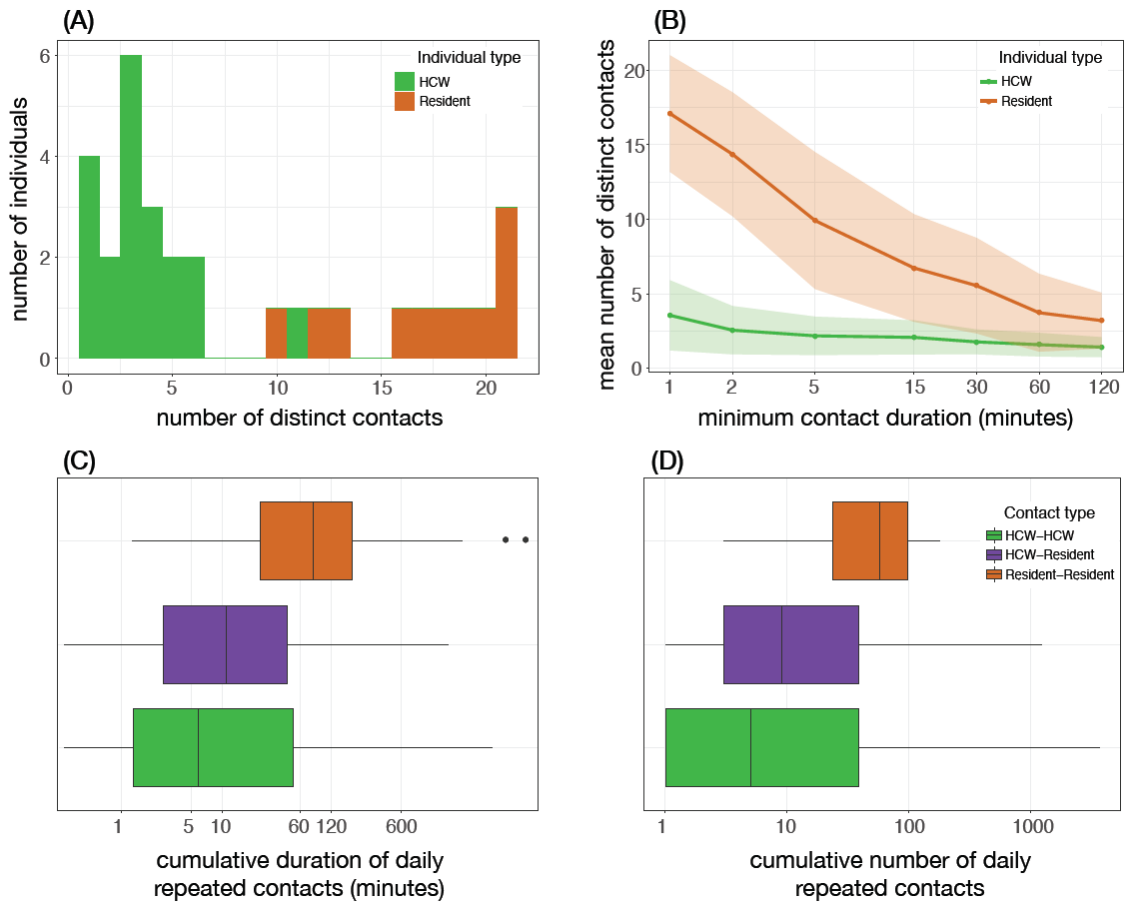
Distinct contacts: Panel (A) illustrates the distribution of the number of distinct contacts (longer than one minute) over the study period, for residents and HCWs; Panel (B) provides the mean (line) number of distinct contacts when varying minimum duration, and one standard deviation (the shaded area) above and below the mean; Panel (C) and (D) represent respectively boxplots for the cumulative duration and frequency of repeated contacts (of duration between 15 seconds and 2 hours), per individual, averaged daily.

We observed a clear dichotomy between residents and HCWs with regards to the number of distinct contacts. During the study period, the distributions of the number of distinct contacts, lasting from 1 minute to 5 hours, marginally overlapped between residents and HCWs (Figure 1A). Residents had a significantly larger number of distinct contacts than HCWs during the study period, especially for short contact durations of 1 to 5 minutes. The mean number of distinct contacts was 3.5 (s.d. 2.3) for HCWs and 17.3 (s.d. 3.6) for residents with contact duration of 1 minute. When varying the minimum contact duration, the difference between the number of distinct contacts of HCWs and residents reduced as the minimum contact duration increased from 1 minute to 2 hours (Figure 1B). The mean number of distinct contacts for residents was also consistently higher than that for HCWs in each day during the study period (Figure S3). Hence, over the study period, the cumulative number of contacts for residents with any individual was substantially larger than the corresponding number for HCWs (Figure S4). We also investigated the repeated contacts that an individual made with other participants in any given day (Figure 1C–1D, Figure S8). For the resident-resident pairs, the repeated contacts (median of 57 repeated contacts with a cumulative duration of 80.6 minutes) was higher than that for the resident-HCW (median 9 repeated contacts and a cumulative duration of 10.9 minutes) or HCW-HCW (median 5 repeated contacts and a cumulative duration of 5.8 minutes) pairs (one way ANOVA *p* = 0.0003). Nearly half of all contacts longer than one minute were among residents (46.6%) and about one third among HCWs (32.7%). The remaining 20.7% were recorded as contacts between residents and HCWs.

### 3.2. Contact distributions

**Figure 2.**
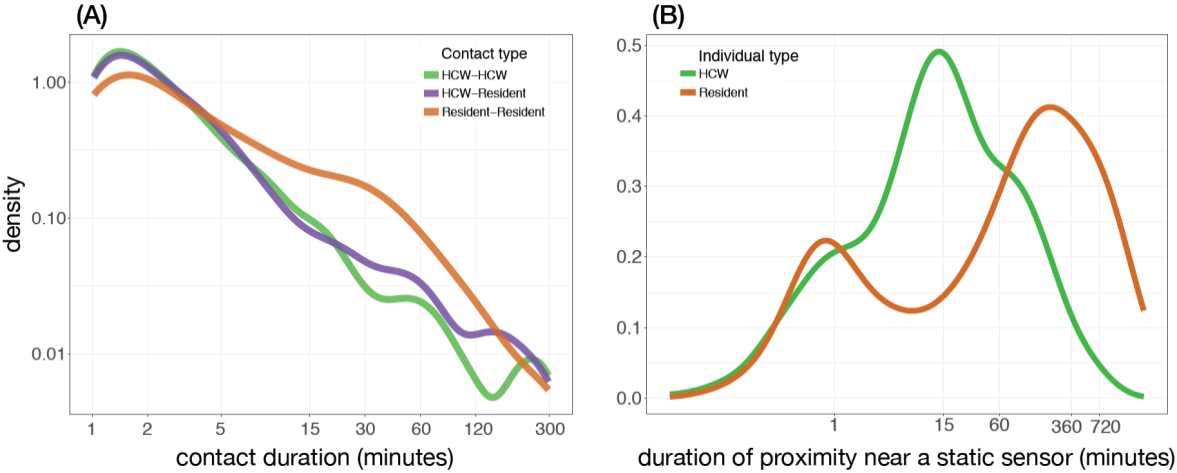
(A) Distribution of contact durations: Estimated kernel density from the empirical distribution of contact durations, by contact pair type. For this plot, only contacts between 6 am and 8 pm, and durations between 1 minute and 5 hours were considered. Both x-axis and y-axis are on the log scale; (B) distribution of time spent close to a static sensor: Estimated kernel density from the empirical distribution of time spent close to a static sensor, averaged over all individuals, during the study period. The x-axis is on the log scale.

The distribution of contact duration for the resident-resident pair was significantly different from the corresponding distributions for resident-HCW and HCW-HCW pairs (Kolmogorov-Smirnov two samples test *p*-value < 10^−15^) (Figure 2A). We did not find a statistically significant difference between the contact duration distributions of resident-HCW and HCW-HCW pairs (Kolmogorov-Smirnov two samples test *p*-value = 0.396). The mean contact duration (of 8 minutes) for the resident-resident pair was longer than that of resident-HCW pair (with mean of 4.6 minutes) or HCW-HCW pair (with mean of 4.2 minutes, respectively). We also observed that the resident-resident pair had a higher frequency of contacts longer than 5 minutes than resident-HCW and HCW-HCW pairs, as illustrated by the larger values of the distribution for long durations (“fat tails”) of the resident-resident distribution in Figure 2A.

The pattern of contacts with respect to the time of the day was dependent on the type of individual pairs considered. The cumulative contact duration averaged across all HCW-HCW contact pairs was the largest and relatively constant over daily shifts between 7 am and 3 pm, and peaked at around 7 pm during weekdays (Figure S5A). The cumulative contact duration averaged across all HCW-resident contact pairs increased rapidly from 9 am and peaked at two different times around 11 am and 4 pm, and then decreased gradually. As expected, evening and overnight (8 pm–9 am) contacts were minimal (Figure S5B). We also observed several intra-day peaks around 8 am, 11 am, 4 pm and 7 pm for resident-resident contacts (Figure S5C), which correspond to breakfast, lunch, snack, and dinner schedules. For signals analyzed here, the hourly patterns for each contact type were evident only when we averaged for the entire study period. However, no specific hourly contact patterns were observable by considering daily signals.

### 3.3. Spatial movements

We analyzed the data for the proportion of time spent by individuals in different locations during daily activities. Residents and HCWs had distinctive movement patterns when considering the duration of time they spent near a static sensor. The distribution of this time duration for HCWs is unimodal, with its mode at about 15 minutes (Figure 2B). However, the corresponding distribution for residents is bimodal, with modes at about 1 minute and 4 hours (Figure 2B). This is expected because the night spent by residents in their rooms (which were equipped with a static sensor) shifts the distribution towards a longer duration.

### 3.4. Clustering

**Figure 3.**
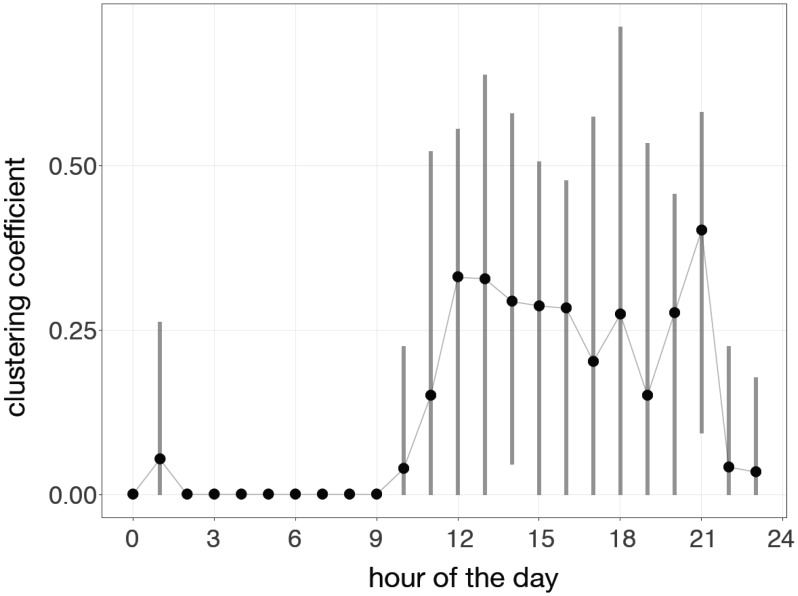
Global clustering coefficient: the network was aggregated in 60-minute segments during the study period. The points represent the hourly mean of the global clustering coefficient and the vertical lines show its extrema within each hour. Only contacts between 1 minute and 5 hours were considered.

We partitioned the study period into segments of 60 minutes and calculated the clustering coefficient on the aggregated contact network during each segment. We observed a diurnal cycle where the clustering coefficient decreased to 0 during night-time from 9 pm to 9 am (Figure 3). The clustering coefficient for this 60-minute snapshot network, considering contacts only during times of activity (i.e., 9 am to 9 pm), was 0.25 (IQR 0–0.42) for the entire study period, which indicates a relatively sparsely connected network. These results were robust when varying the length of time segment for calculating the clustering coefficient (Figure S8).

## 4. Discussion

In this study, we presented an analysis of movement data to infer the interactions between individuals and their environment in the largest veterans LTCF in Canada. Our observations show a dichotomy between the contact network and movement patterns of residents and healthcare workers in this facility (Figures 1 and 2). Overall, we found that residents tend to contact more individuals, for a longer duration, and are less mobile than healthcare workers. We estimated the time-varying global clustering coefficient of the contact network in this facility, which has a moderate—but highly variable—value during the day between 9 am and 9 pm (Figure 3). Hence, we do not infer the interactions as a tightly interconnected network (on average), but our analysis of contact patterns presented here suggests a highly structured network (between residents and HCWs) in this facility.

The detailed knowledge of contact events allows for the re-creation of an artificial in-silico population to simulate potential disease spreading phenomena with a high degree of realism. It is also possible to test, in simulations, the impact of disease-specific interventions for mitigation and containment. Highly detailed contact data might, however, represent unnecessarily detailed information if the goal is to simply extract stylized facts or generic statistics of contacts, which can be used to design and validate models of human contacts and to estimate the relevance of transmission control strategies.

The observed sequence of contacts may be influenced by specific aspects of the environment in which the measure takes place, while representing only one instance of many possible contact sequences that can occur in the same environment at different times. It is thus useful to build contact summary statistics, which are expected to be more robust to variations of the specific measurements. To this end, the analysis of time-dependent contact data are usually aggregated along spatio-temporal dimensions, as discussed and analyzed in our study. Temporal aggregation yields cumulative contact networks that preserve the information at the individual level. Such networks describe which tags have been in contact, considering that each link between two nodes is weighted by the cumulative time spent in contact by the two interacting individuals. The heterogeneity of contact patterns at the individual level is known to have a strong impact on disease spread dynamics [12]. In particular, it highlights the existence of “super-contactors”, i.e., individuals who account for a significant proportion of the overall contact durations and may therefore become super-spreaders in the case of an infectious disease outbreak [13]. Spatial aggregation allows to disentangle the multitude of individuals' movements and to identify patterns at the group level. Here, we observed that HCWs were more mobile than residents. Spatial movements can also inform the extent of environmental contamination (e.g., pathogens shedding on fomites and surfaces like door knobs, beds, floors, and tables).

The results presented here may not be directly extrapolated to other long-term care facilities. Indeed, the size, structure, design and work policies can vary among healthcare facilities. However, our analysis highlights the importance of context-specific data that are essential to inform models and support epidemiological, clinical, and public health decisions. In particular, the various summary statistics and distributions presented here will be useful to parameterize mathematical models simulating disease transmission dynamics in similar facilities. Such simulation studies often lack relevant data to validate their models and outcomes. In the context of disease spread in healthcare facilities, both direct (i.e., contacts between individuals) and indirect routes (i.e., contaminated environment) can be important, and therefore knowledge of contact and movement patterns of individuals plays an important role in clinical decision-making and implementation of infection control practices. For example, we have recently evaluated the impact of intervention measures on nosocomial influenza [8]. Parameterizing an agent-based model of disease spread with movement data in the LTCF studied here, we have shown that the largest reduction of attack rates (i.e., the fraction of at-risk population infected throughout the outbreak) is achieved when residents were offered antiviral prophylaxis for the duration of the outbreak. In contrast to this prediction for data-driven movements, the lowest attack rates under the assumption of random mixing were observed when the isolation of residents with symptomatic infection was implemented. These findings indicate that the strategy outcomes are highly dependent on the contact patterns in the particular facility [8].

Our study identified a number of technical limitations that appeared only at the stage of data analysis. For example, we identified and filtered out a significant amount of interferences between devices and readers. However, determining all the noise in a database can be a difficult task (if not impossible), which is also the case in our datasets. We identified unexpected patterns in the data that may have been caused by interferences between individuals' sensors and static tags. For example, we speculate that some signals received from rooms located in the middle of the study area was transferred by the static readers from the neighboring rooms (Figure S6). However, we were not able to ascertain whether such signals were noise or represented true contacts. Therefore, we included them in our analysis. Furthermore, as for most data collection studies involving individuals' behaviour, the relevance of our analysis may depend on the participation rate. Our analysis here is based on a relatively low participation rate of 36% for residents, and may have missed important contact and geolocalization information regarding the non-participants group (e.g., contacts with and within this group). However, even with high participation rates, the incorrect use of the tracking technology can reduce the quality of data and their representation of the real connectivity during individual movements.

Our experience of data collection and analysis has provided us with important insights that need to be considered in future studies. First and foremost is the fact that radio signals have propagation characteristics such as being absorbed by some materials (e.g., water and body mass), passing through some materials (e.g., glass), and being reflected by various metals. These are in addition to sources of electromagnetic radiation that may interfere with RFID devices. As a result, it is possible to detect seemingly “random” contacts over a long distance, as well as occasional “blind spots” contacts occurring. It is therefore important to develop appropriate analysis tools and modelling frameworks that are robust to missing data and outliers. It should also be noted that infrastructure deployment and protocols of the candidate facilities can play a key role in both participation rates and quality data. Finally, we recommend that studies of this scale within healthcare facilities should pair RFID technologies with other means of data collection such as observational studies in order to validate the system dynamics and analysis of outcomes. Despite these technological limitations, Internet of Things and in particular RFID based systems remain an inexpensive and scaleable data collection strategies, when compared to direct observations. These properties are particularly important for longitudinal studies and those involving a large number of participants, while featuring potential for more objective measures than traditional, non-automated methods of survey.
