# Supplementary material for: Individual movements and contact patterns in a Canadian long-term care facility
Source: AIMS Public Health. 2018 May 9;5(2):111–21. doi: 10.3934/publichealth.2018.2.111 (PMC6079054; doi:10.3934/publichealth.2018.2.111)
Supplement: Supplementary file 1 [file publichealth-05-02-111-s01.pdf]

Public Health

AIMS Public Health, 5(2): 111–121.
DOI: 10.3934/publichealth.2018.2.111
Received: 29 November 2017
Accepted: 07 May 2018
Published: 09 May 2018

http://www.aimspress.com/journal/aimsph

Research article

Individual movements and contact patterns in a Canadian long-term care facility

David Champredon[1], Mehdi Najafi[2], Marek Laskowski[1,3], Ayman Chit[4] and Seyed M. Moghadas[1,*]

[1] Agent-Based Modelling Laboratory, York University, Toronto, ON M3J 1P3, Canada
[2] Department of Mechanical & Industrial Engineering, Faculty of Applied Science & Engineering, University of Toronto, Toronto, ON M5S 3G8, Canada
[3] Schulich School of Business, York University, Toronto, Ontario, Canada M3J1P3, Canada
[4] Sanofi Pasteur, Swiftwater, PA, USA, and Leslie Dan Faculty of Pharmacy, University of Toronto, Toronto, ON M5S 3G8, Canada

* **Correspondence:** Email: moghadas@yorku.ca.

Supplementary material

This supplementary material presents details of processing on a large dataset that was recently collected using “Open-Beacon” wearable proximity sensors. This dataset, analyzed in the main text, contains signals of face-to-face interactions of individuals within a long-term care facility. Here, we provide more details about the methodology used to determine the spatial location for individuals wearing the wireless devices.

Data collection and processing

The data collection infrastructure was based on wearable wireless devices that exchange radio packets in a peer-to-peer fashion to monitor the location and proximity of individuals. Use of ultra-low-power radio signals allows radio packets to be exchanged only between devices located within a specified distance, in this case being 1.5 meters. Similar to Cattuto and et al. [1], our system detects and records close-range meetings during which a communicable disease infection could be transmitted, e.g., by coughing, sneezing, or hand contact [2]. The radio frequency

identification (RFID) readers submitted information received from the tags under coverage to a data server for further analysis (Figure S1).

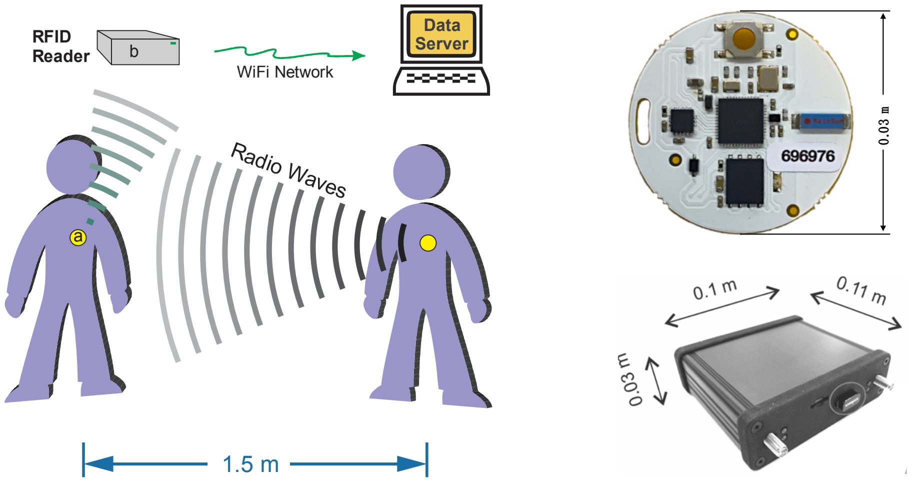

Figure S1. The schematic working model of the RFID tags using radio signals and data flow with a 3-cm diameter wearable tag, which sends signals captured by an RFID reader in time units of mili-seconds.

The sensing system was tuned so each contact between participants was detected and recorded, with a temporal resolution of milliseconds. It is therefore possible to determine the number of contacts that each individual established with any other individual, the duration of individual encounters, the cumulative time spent in contact between two or more individuals, the frequency of encounters, and how these quantities evolve during the study period.

In order to estimate the location of an individual, a set of routines is used as described below. Localization techniques fall into two categories, namely, range-based and range-free positioning. Range-based localization commonly implemented through two main techniques: Distance-based and angle-based methods. Distance-based localization depends on measuring the distances between the node to be positioned, and at least three reachable anchors (i.e., known positions) in case of 2-dimensional positioning systems, which is called *Trilateration*. Angle-based localization depends on measuring the angles between the nodes to be positioned with at least three reachable anchors; this approach is known as *Triangulation*. The second category, which is range-free, is independent of any distance or angle estimations, instead, it depends on different techniques such as ring overlapping or triangle overlapping [3]. Localization using wireless sensor networks does not provide the actual position of a node, but it provides the opportunity to estimate the positions. In doing so, several parameters can be used to estimate distances between nodes, including Received Signal Strength Indicator (RSSI), Time of Arrival (TOA), Time Difference of Arrival (TDOA), Round Trip Time of Arrival (RTTOA) and Radio Hop Count (RHC) [3].

In this study, the RSSI value (determined when a reader detects signals from a tag) has a reverse relationship with the distance of the tag from the reader. Since the location of the readers were fixed and known, a relative distance of the tag and the reader in a measurement of signal strength could be identified. It should be noted that, the location of an individual with a tag was identified through the signals received, indicating to have a close contact (or an edge) with the marker tag mounted at that

location. We also used fine locating algorithms based on geometrical modelling and discretization of the entire long-term care facility. More than one marker tag was used in corridors and areas that have complex physical structures. Since the range of a marker tag radio signal is always larger than the extent of the assigned location, an individual tag would be recognized to be in more than a location at the same time. Thus, we utilized geometrical information in the form of a grid model to locate individuals accurately and avoid multiple-location interpretations. To this end, we built a radio space considering the locations of fixed marker tags as anchors and used trilateration algorithm to estimate the locations of moving tags following the method described in previous studies [4,5].

Additional results

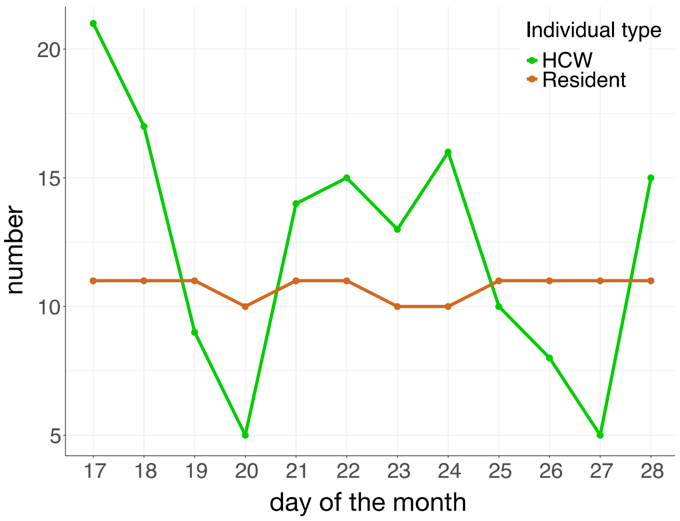

Figure S2. Number of sensor tags of distinct individuals recorded daily from March 17 to 28th 2016. Saturdays and Sundays were on March 19th, 20th, 26th and 27th, 2016.

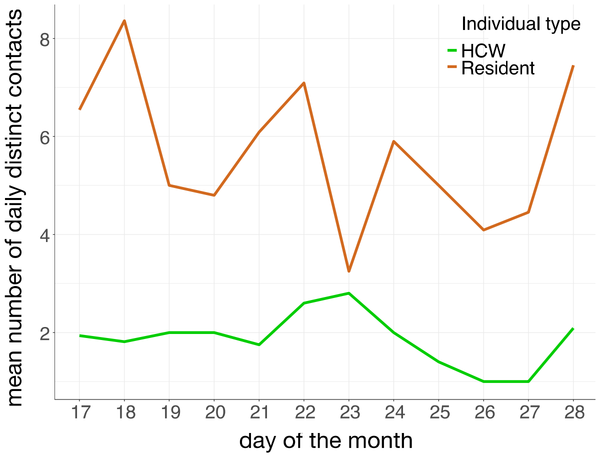

Figure S3. Mean number of daily distinct contacts (longer than 1 minute).

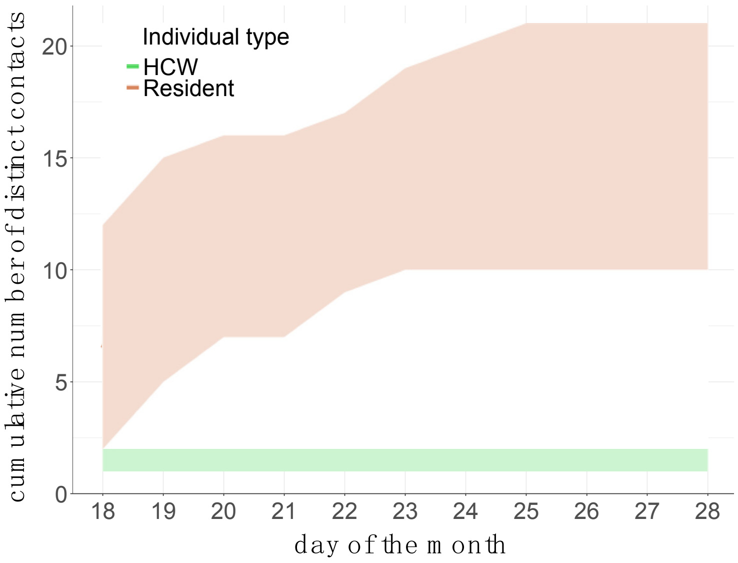

Figure S4. Cumulative number of distinct contacts recorded during the study period. The line represents the mean number and the shaded area shows the extrema across all individuals of each type (i.e., resident or HCW).

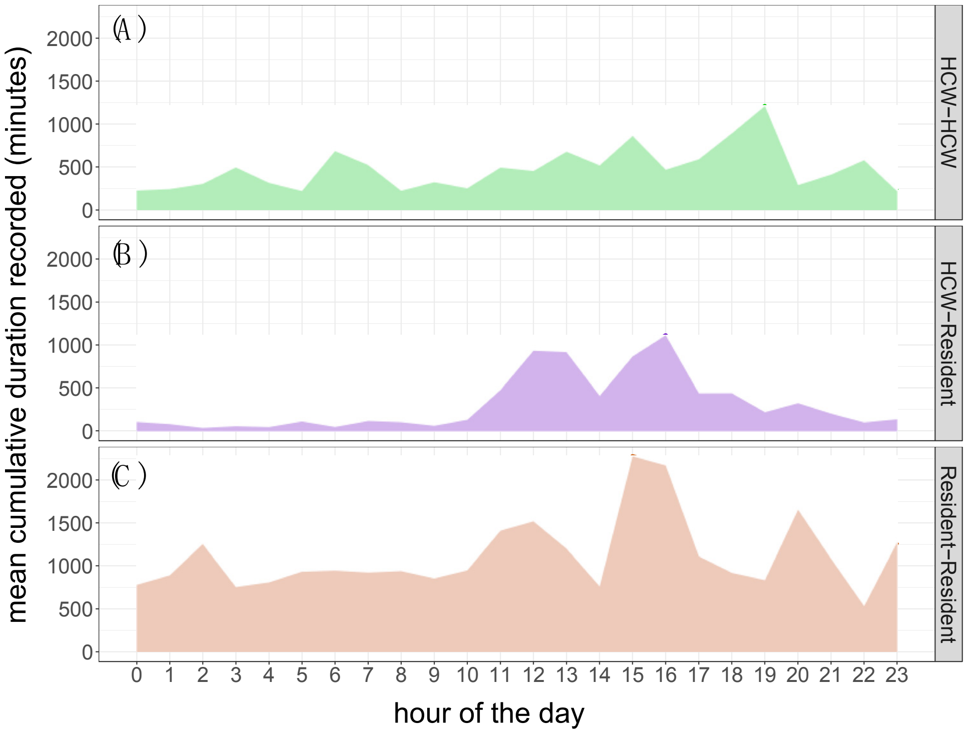

Figure S5. Hourly pattern of individual contacts. The cumulative duration of contacts recorded between individuals was averaged over the study period, by hour of the day and individual contact pair type. Only contacts between 1 minute and 5 hours were considered.

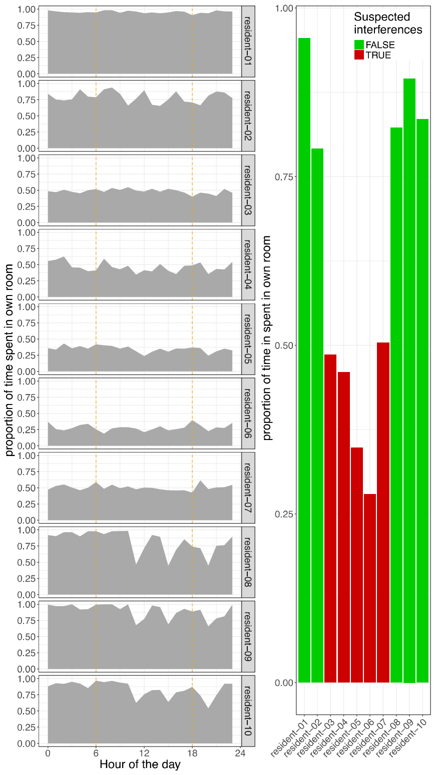

Figure S6. Proportion of time spent by residents in their own rooms and suspected interferences. The resident numbers are ordered according to their spatial location along the main corridor in the facility. “resident-01” lived in the room located at the northern end of the facility, whereas “resident-10” was at the most southern end. The proportion of time was averaged for each hour of the day during the study period.

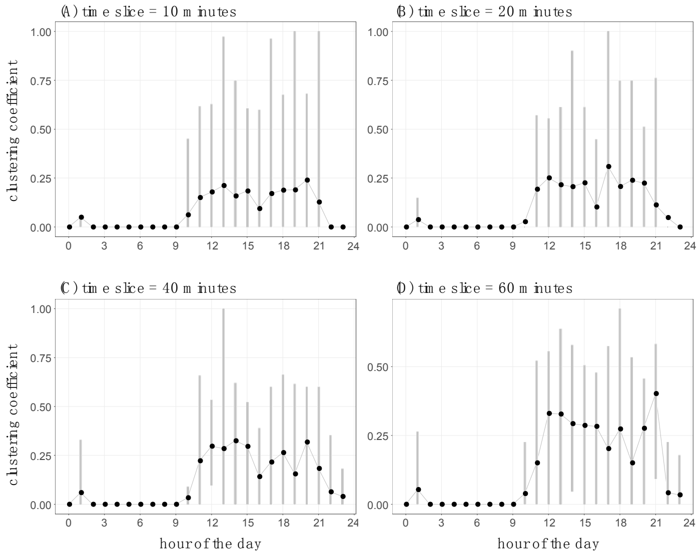

Figure S7. Sensitivity analysis for the dynamics of the clustering coefficient with respect to the time segment chosen for aggregation.

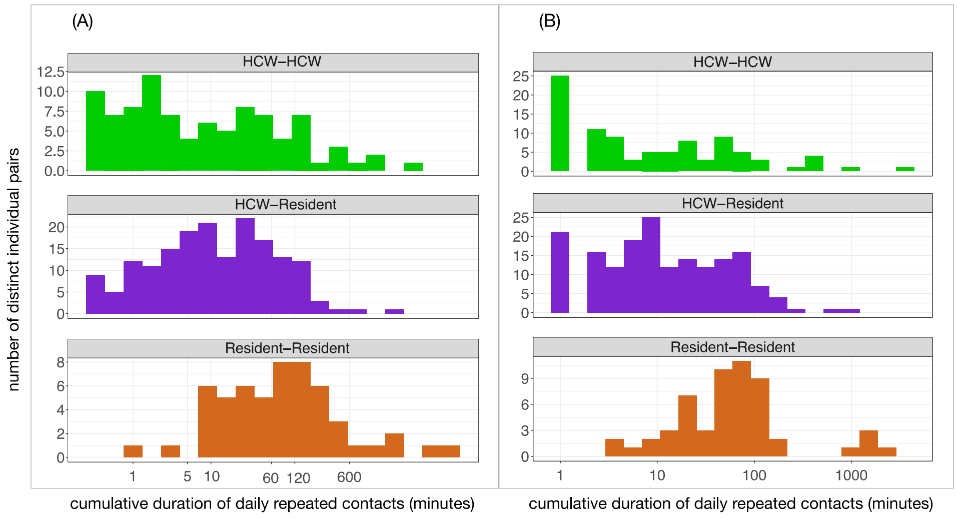

Figure S8. Distribution of the cumulative duration (Panel A) and frequency (Panel B) of repeated contacts (of duration between 15 seconds and 2 hours), per individual, averaged daily. Note that the x-axis is on a log-scale.

References

1. Cattuto C, Broeck WVD, Barrat A, et al. (2010) Dynamics of person-to-person interactions from distributed RFID sensor networks. *PloS One* 5: e11596.
2. Vanhems P, Barrat A, Cattuto C, et al. (2013) Estimating potential infection transmission routes in hospital wards using wearable proximity sensors. *PloS One* 8: e73970.
3. Hightower J, Borriello G (2001) A survey and taxonomy of location systems for ubiquitous computing. *IEEE Comput* 34: 57–66.
4. Saxena M, Gupta P, Jain BN (2008) Experimental analysis of RSSI-based location estimation in wireless sensor networks. *Int Conf Commun Syst Software Middleware Workshops* 2008: 503–510.
5. Moore D, Leonard J, Rus D, et al. (2004) Robust distributed network localization with noisy range measurements. Proceedings of the 2nd international conference on Embedded networked sensor systems 2004 Nov 3, 50–61, ACM.

© 2018 the Author(s), licensee AIMS Press. This is an open access article distributed under the terms of the Creative Commons Attribution License (http://creativecommons.org/licenses/by/4.0)
